# Correction: Neubert et al. Effects of Normobaric Hypoxia and Adrenergic Blockade over 72 h on Cardiac Function in Rats. *Int. J. Mol. Sci.* 2023, *24*, 11417

**DOI:** 10.3390/ijms25158408

**Published:** 2024-08-01

**Authors:** Elias Neubert, Beate Rassler, Annekathrin Hoschke, Coralie Raffort, Aida Salameh

**Affiliations:** 1 Carl-Ludwig-Institute of Physiology, University of Leipzig, 04103 Leipzig, Germany; elias-neubert@gmx.de (E.N.); anne.hoschke@web.de (A.H.); 2 Department of Pediatric Cardiology, Heart Centre, University of Leipzig, 04289 Leipzig, Germany; coralieraffort@orange.fr (C.R.); aida.salameh@medizin.uni-leipzig.de (A.S.)

In the original publication [1], there was a mistake in Table 3 as published. The table contained some identical values in several groups in some lines of Table 3. The corrected Table 3 appears below. The authors state that the scientific conclusions are unaffected. This correction was approved by the Academic Editor.

## Figures and Tables

**Table 3:** Hemodynamic results.

Cohort	Normoxic Cohort:				Hypoxic Cohort:			
Group (*n*)	N-Ctrl (9–14)	N-PZ (6–7)	N-PR (6–8)	N-PZ + PR (6–8)	H-Ctrl (14–16)	H-PZ (9–13)	H-PR (7–12)	H-PZ + PR (9)
EF [%]	**56.9 ± 1.8**				**48.8 ± 1.7** ##			
	61.8 ± 1.2	62.6 ± 1.5	49.5 ± 5.6 *	50.7 ± 4.3 *	54.1 ± 2.9 #	45.9 ± 2.5 ## *	42.7 ± 4.2 *	48.4 ± 3.4
SW [mmHg µL]	**15,001 ± 1146**				**10,954 ± 664** ##			
	18512 ± 2174	16857 ± 1104	12476 ± 1560 *	10467 ± 1532 ***	12852 ± 1513 ##	9577 ± 939 ##	9151 ± 1164	11830 ± 1095
HR [min^−1^]	**426.0 ± 7.7**				**390.0 ± 6.2** ###			
	441.8 ± 8.1	458.1 ± 11.2	423.9 ± 9.3	374.5 ± 21.1 ***	410.4 ± 8.3 #	407.4 ± 11.1 ##	359.6 ± 11.9 ### ***	367.7 ± 13.9 **
LV esE [mmHg/µL]	**0.33 (0.26; 0.50)**				**0.36 (0.24; 0.71)**			
	0.36 (0.24; 0.53)	0.28 (0.26; 0.34)	0.44 (0.37; 0.55)	0.24 (0.17; 0.50)	0.34 (0.26; 0.66)	0.57 (0.28; 0.76)	0.33 (0.25; 0.58)	0.25 (0.18; 0.72)
RV dP/dtmax [mmHg/s]	**2053 (1602; 2488)**				**1919 (1530; 2687)**			
	2154 (2072; 2448)	2441 (2001; 3233)	1888 (1609; 3348)	1473 (1390; 1719)	2511 (1823; 3118)	2238 (1799; 3049)	1568 (1503; 1919)	1402 (1158; 1631) *
RV dP/dtmin [mmHg/s]	**−1711 (−2145; −1215)**				**−1618 (−2138; −1274)**			
	−1896 (−2501; −1727)	−1863 (−2164; −1166)	−1409 (−1774; −1228)	−1208 (−1454; −1053)	−2088 (−2384; −1487)	−1856 (−2291; −1236)	−1379 (−1681; −1077)	−1333 (−1778; −1098)
MAP [mmHg]	**96.4 ± 3.5**				**91.0 ± 2.4**			
	109.6 ± 3.5	91.0 ± 5.7	92.7 ± 11.6	84.7 ± 5.0	91.4 ± 3.6	91.2 ± 4.8	87.1 ± 5.9	95.2 ± 5.7
TPR [mmHg min kg s^−1^]	**0.28 (0.25; 0.33)**				**0.31 (0.29; 0.41)** #			
	0.28 (0.25; 0.32)	0.25 (0.24; 0.28)	0.28 (0.23; 0.46)	0.31 (0.26; 0.38)	0.30 (0.25; 0.33)	0.30 (0.27; 0.46)	0.33 (0.30; 0.43)	0.40 (0.30; 0.51)

Normally distributed data are given as means ± SEM; if data were not normally distributed, the medians (25th; 75th percentiles) are presented (*n* = number of values per group). Cohort means (± SEM) and medians (25th; 75th percentiles) are presented in bold characters. Cohort/group labels: N = normoxia; H = hypoxia; Ctrl = control group (0.9% saline infusion); PZ = prazosin; PR = propranolol. LV, RV = left/right ventricle; EF = ejection fraction; esE = end-systolic elastance; SW = stroke work; dP/dt max = maximal velocity of contraction; dP/dt min = maximal velocity of relaxation; HR = heart rate; MAP = mean aortic pressure; TPR = total peripheral resistance. Significant differences from the hypoxic cohort or groups to the normoxic cohort or to the corresponding normoxic group: # *p* < 0.05; ## *p* < 0.01; ### *p* < 0.001; significant differences between blocker groups and the related control (N-Ctrl or H-Ctrl) are marked with asterisks: * *p* < 0.05; ** *p* < 0.01; *** *p* < 0.001.
